# Design and prototype of TOMO: an app for improving drug resistant TB treatment adherence

**DOI:** 10.12688/f1000research.67212.1

**Published:** 2021-09-29

**Authors:** Anis Fuad, Guntur Budi Herwanto, Ariani Arista Putri Pertiwi, Siska Dian Wahyuningtias, Harsini Harsini, Ahmad Watsiq Maula, Diyah Utami Kusumaning Putri, Ari Probandari, Riris Andono Ahmad

**Affiliations:** 1 Department of Biostatistics, Epidemiology and Population Health, Faculty of Medicine, Public Health, and Nursing, Universitas Gadjah Mada, Yogyakarta, Yogyakarta, 55284, Indonesia; 2 Center for Tropical Medicine, Faculty of Medicine, Public Health and Nursing; Universitas Gadjah Mada, Yogyakarta, Yogyakarta, 55284, Indonesia; 3 Department of Computer Sciences and Electronics, Faculty of Mathematics and Natural Sciences, Universitas Gadjah Mada, Yogyakarta, 55284, Indonesia; 4 Department of Basic and Emergency Nursing, Faculty of Medicine, Public Health and Nursing, Universitas Gadjah Mada, Yogyakarta, Yogyakarta, 55284, Indonesia; 5 Department of Pulmonology, Dr. Moewardi General Hospital, Surakarta, 57126, Indonesia; 6 Department of Public Health, Faculty of Medicine, Universitas Sebelas Maret, Surakarta, 57126, Indonesia

**Keywords:** m-health, tuberculosis, drug resistance, medication adherence, smartphone, Indonesia

## Abstract

**Background:** Drug resistance and multi drugs tuberculosis (DR/MDR-TB) are associated with patients’ low adherence to undergoing complex treatment. Driven by the increasing use and penetration of a smartphone and the End of TB Strategy that seeks for digital health solution, Center for Tropical Medicine Universitas Gadjah Mada has developed TOMO, an Android-based app for improving medication adherence in MDR-TB.

**Objective:** This paper aims to present the sequential steps to develop the app, its general architecture, and its functionalities.

**Methods:** It is a design thinking process involving two MDR-TB referral centers, district health offices, primary health centers, and MDR-TB patients in Central Java and Yogyakarta, Indonesia. We adopted the Principles for Digital Development to develop and design the app. MDR-TB treatment guideline from the Indonesian Ministry of Health was used to develop functionalities of the app for improving adherence.

**Results:** TOMO app could be used by patients, primary health centers, clinical teams, and case managers. The app prototype features include adverse event records and reports, medication-taking reminders, and communication between the patient and the TB-MDR case manager. We have successfully tested the functionalities based on four use cases: patients with high adherence, patients with low adherence, patients with adverse events, and patients following treatment in the primary health center without any visit to the MDR-TB center.

**Conclusion:** TOMO app has contributed to the limited body of literature on improving TB-MDR adherence with digital health intervention, especially using a health app. The app has been tested using four scenarios. We will follow up with usability testing before implementing the app in a real setting.

## Introduction

Multi-drug resistant tuberculosis (MDR-TB) is associated with high treatment failure due to low adherence of patients in` undergoing the complex treatment process. ^1^ The prolonged duration of treatment, the huge opportunity costs incurred by daily visits to health facilities, and the wide range of adverse drug events have been associated with high default rates of MDR-TB patients. ^1^ Stigma, feeling ashamed, and boredom are also huge barriers to improving patient adherence to treatment. ^2– 4^

In Indonesia, the country with the fourth largest tuberculosis (TB) burden, MDR-TB cases are alarming. It is estimated that there are 32,000 cases of MDR-TB of which only 8% were detected in the TB program. The performance of MDR-TB case management urgently needs to be improved. In 2016, about 29% of cases died within the first six months after diagnosis, and 19% did not continue treatment. Therefore, a patient-centered care approach is necessary to support MDR-TB patients during long treatment to ensure medication adherence, oversee potential side effects, and offer psychosocial support. Adherence to the long course of TB-MDR treatment is a complex endeavor involving multiple factors that influence patients’ motivation and behavior in treatment-taking. ^1, 5, 6^

Previous studies on other chronic conditions have shown that treatment adherence can be improved using m (mobile)-health. ^6– 10^ One of the functions of m-health is to empower patients by providing information for decision-making, consultation and advice from physicians, treatment regimen schedules, and providing better opportunities to control their healthcare. ^11– 14^ In developing countries, m-health is mainly used for providing disease information, reminders for care, and telemedicine. However, few m-Health applications exist for TB. Iribarren et al. (2016) found only two apps, and none were related to TB treatment self-management or patient-provider interaction. ^15^

Digital health technologies have been considered a potential tool to improve medication adherence in tuberculosis. ^9, 14^ Through mobile health innovations, patients will be empowered to easily request consultation and advice regarding treatment regimen, scheduling, and side effects. A recent study exploring patients and medical staffs in six countries reported a high enthusiasm for e-health innovation to support TB programmes, including apps ^16^ and video observing treatment apps. ^17^

Digital health intervention has been recommended by WHO to strengthen health systems in at least nine use cases. ^18^ Digital health intervention is projected to contribute to substantial cost saving and improvement of TB programs. ^13, 19^ A comprehensive review of the potential for digital health in TB programmes has been published. However, there are still only a few studies that attempt to explain the role of digital health innovation in improving medication adherence.

Along with the increasing ownership of cellular phones, especially smartphones, health intervention through applications is no longer just a reminder or reciprocal communication. Health apps can also act as an integrated application that includes various functions developed for specific purposes. In this context, we intend to report on the design and technical overview of apps specifically designed to help manage the MDR-TB program.

This paper aims to:

1. Describe sequential steps to develop apps involving multiple stakeholders in the MDR-TB program
2. Draw technical architecture and interoperability of the app with the existing MDR-TB information system
3. Test features and functionalities of the app based on scenarios in the real setting.

## Methods

This study was a collaborative work by the Center for Tropical Medicine Universitas Gadjah Mada, Dr. Sardjito hospital in Yogyakarta province, Dr. Moewardi hospital in Central Java province, and Computer Science department of Universitas Gadjah Mada. Following subsequent steps in design thinking, all collaborators teamed up to develop the best health app solution to improve MDR-TB treatment adherence.

Participants in this study were patients with MDR-TB in Dr. Moewardi hospital, healthcare professionals (pulmonologists from Dr. Sardjito and Dr. Moewardi hospital, MDR-TB case manager at hospital and primary health care level), and district health department officers. Researchers from the Center for Tropical Medicine Universitas Gadjah Mada were the principal investigators in this study and translating user needs from clinical setting perspectives into health app workflow and design.

## Study design

Five steps of design thinking were adopted in this study; empathize, define, ideate, prototype, and test. ^20^ Design thinking is an analytic and creative approach in solving problems or fulfilling user needs in the app development context. ^21, 22^ Perspectives from multiple stakeholders or users that will use the health app were the most important data in the app development processes. The details of each step and type of data collected are depicted in Table 1.

**Table 1.** Prototype development process.

Step	Activity	Objective	Participant	Data
Empathize	Discussion with pulmonologists	To gain insight into MDR-TB treatment workflow in the hospital setting To identify needs and challenges in MDR-TB treatment adherence	Pulmonologists in two hospitals (Dr. Sardjito and Dr. Moewardi)	Meeting minutes and summaries
	Observation of MDR-TB treatment procedures in Dr. Moewardi hospital MDR-TB clinic	To gain insight into the current MDR-TB treatment workflow To identify problems faced by the MDR-TB case manager	MDR-TB case manager Patients with MDR-TB	Observation report
	Observation and analysis of existing MDR-TB app (eTB Manager)	To identify any limitations and possible data interoperability of existing app	MDR-TB case manager Pulmonologist Researcher	Observation report
	Survey of MDR-TB patient needs and expectation	To identify user needs and challenges	MDR-TB patients	Survey data
Define	Workshop 1	To summarize the needs and challenges of each user	Researcher Pulmonologist App developer team	Workshop minutes and summaries
Ideation	Workshop 2	To list and choose possible and feasible solutions (app workflow and design)	Researcher Pulmonologist Case manager App developer team	Workshop minutes and summaries
Prototype	UI/UX development and trial	To get initial feedback on the app workflow and design	Researcher Pulmonologist MDR-TB case manager App developer team	UI/UX file Feedback notes
	App development process	To create app prototype based on defined workflow and design	Researcher App developer team	App architecture document Source code
Test	App simulation	To identify app performance in a daily setting	Researcher App developer team	Simulation log
	Workshop 3	To introduce, test, and collect feedback from users on app performance	Researcher Pulmonologist MDR-TB case manager (hospital and primary health care) App developer team	Workshop minutes and summaries
	Workshop 4	To introduce, test, and collect feedback from users on app performance	Researcher MDR-TB case manager (hospital and primary health care) MDR-TB patient App developer team	Workshop minutes and summaries

We conducted meetings with two MDR-TB referral hospitals; Dr. Sardjito hospital in Yogyakarta province and Dr. Moewardi hospital in Central Java province. The first meeting with Dr. Sardjito hospital icluded the empathize and define processes to collect and summarize any stories and problems that providers had in monitoring MDR-TB treatment. In addition, this study also collected information from MDR-TB patients to ensure the app covered all user needs. After problems were defined, the ideate process was started by creating a list of possible solutions and the workflow of TB application features. The prototype process based on the chosen solution was conducted iteratively. Dr. Moewardi hospital was used as the primary setting to improve the workflow of the TB app. Two workshops to introduce the app for case managers, physicians, and 13 PHC in Dr. Moewardi’s area were conducted in the test process. Prior to the workshop, a simulation mimicking use cases in a real setting was conducted by researchers.

In 2017-2018, Dr. Moewardi hospitals provided treatment to 207 patients. MDR-TB clinic in this hospital is supported by one pulmonologist, one dedicated case manager, five other supporting staff. This hospital utilized TB-Manager, a web-based application from the Ministry of Health, to register, monitor, and report the MDR-TB program. Later, this program was changed with a new electronic system called SITB (Sistem Informasi TB). For internal purposes, the hospital also implemented a hospital information system for billing and recording all patients’ transactions, including MDR-TB.

## Ethical considerations

This study was started in September 2019. The study protocol and procedure were approved by the Ethical Committee of the Faculty of Medicine, Public Health and Nursing, Universitas Gadjah Mada, Yogyakarta (No: KE/FK/0990/EC/2019 and KE/FK/1285/EC/2020).

## Results

A total of four workshops and seven other activities, including observation, survey, and simulation, were conducted in this study.

***Insight from the empathize and define steps***

- The MDR-TB treatment monitoring using the current system was not able to track patient treatment adherence daily. Furthermore, the system was only focused on collecting patients baseline data and drug regimens.
- Primary health care performance in supervising dai drug consumption of MDR-TB patients was not monitored. Primary health care in MDR-TB treatment is vital since patients need to visit it daily to take the drugs and report any adverse events.
- Communication activities related to adverse events of MDR-TB drugs were not well organized and documented. Patients could directly consult the pulmonologist, or sometimes they could ask the case manager or primary health care first. During the consultation, the information was not recorded and could cause problems for further patient care related to the adverse events.
- Adverse drug effects were issues that influenced patient adherence to MDR-TB treatment. In addition, the patients also needed social support and opportinuies to share amongst patients with MDR-TB to improve treatment adherence.
- The findings from the patient’s survey were that most patients owned an Android smartphone (69.23%), and that around 80% of respondents used internet browsers and social media apps (i.e., Whatsapp).

***The solution identified from the ideation step***

- In workshop 2, users, researchers, and the app development team decided on solutions that would become the app’s main functionalities. The first function was reporting MDR-TB drug consumption for patients and cross-checking the patients’ drug consumption by healthcare workers in the hospital or primary health care. Second, a drug adverse event reporting function could be accessed by pulmonologists, case managers, and primary healthcare workers. Third, a real-time dashboard to monitor the treatment progress of TB-MDR patients.
- In order to be interoperable with the national TB information system, the research team has discussed with the TB program manager and the technical team to develop an Application Programming Interface (API), so that data from the two systems can be exchanged securely without user intervention.
- A MDR-TB patient forum within the app could help patients give social support to each other during treatment. Post-treatment patients could also share their experiences to help other patients who are still undergoing the treatment process.

In the ideation step, some vital app features were chosen for initial prototype development. The cross-checking drug consumption, drug adverse event report and consultation, and monitoring dashboard were chosen for the initial phase. Other important features will be developed later after the prototype’s main features are implemented in the real setting.

## Prototype and test step

The User Interface (UI) and User Experience (UX) were developed to depict the initial app workflow and design. After several tests and revisions by researchers and users (pulmonologists and the case manager), the technical architecture and workflow of the app were established.

### The technical architecture of TOMO

**Figure 1.**
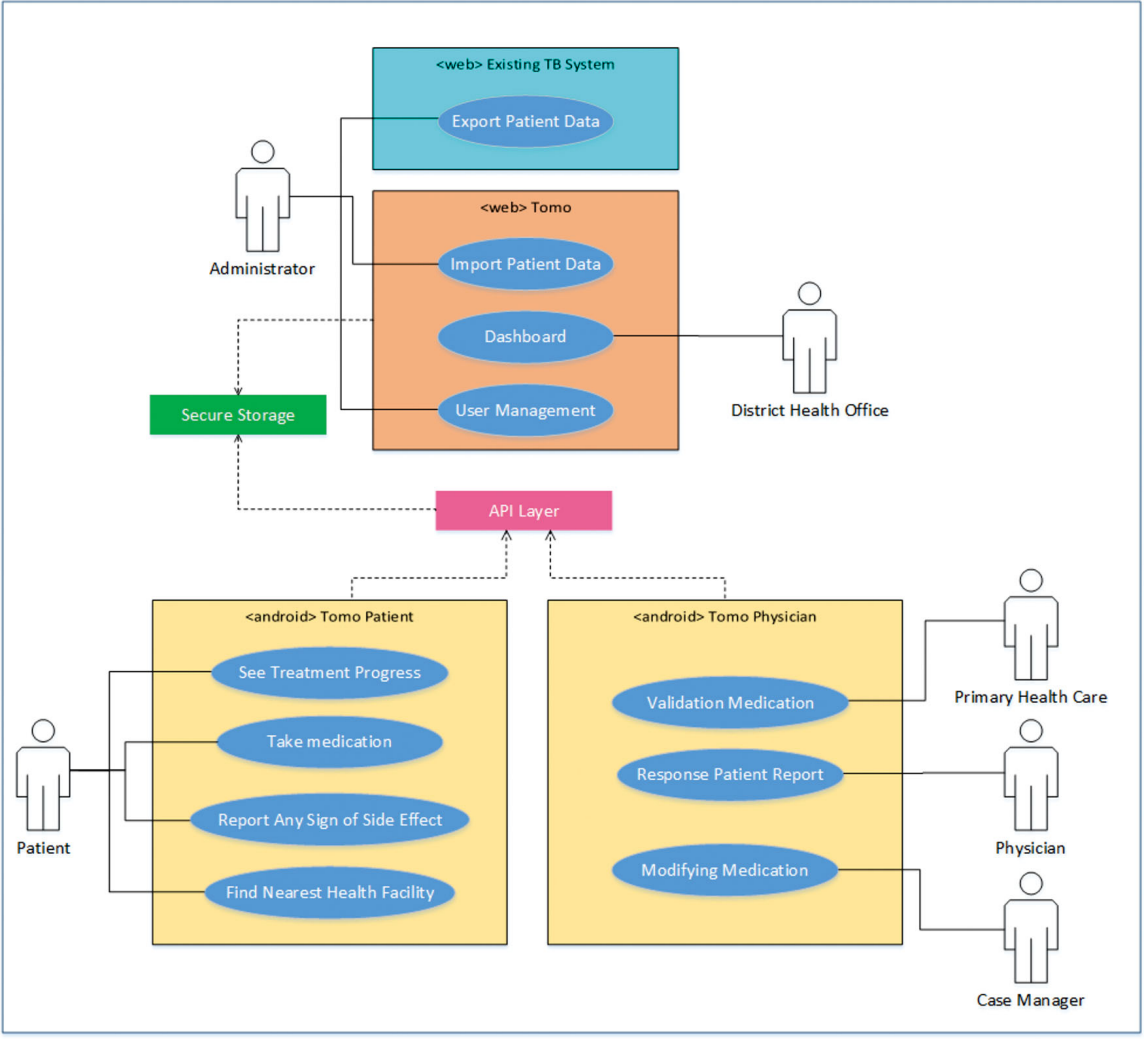
Use case of TOMO.

The first application is for TB-MDR patients (TOMO). The second application is dedicated only to providers, including Primary Health Centers, the case manager, and the treating physician (TOMO CM). The first two applications are targeted as mobile-based applications. Before publication of this paper, we will have developed the Android application to be available on PlayStore. The third application is a web-based application that will be used as a dashboard by the government to see the usage overview of the application. The web-based application is also used as an admin tool for managing user and master data. These three applications work together to achieve the primary goal. Figure 1 shows the use diagram that describes the functionality of each user within the system.

The current prototype of the TOMO application was developed in an Android environment using Kotlin programming language. Furthermore, the TOMO website was developed using PHP 7 programming language. MySQL 5.6 was used as the database management system, while Django Python (Django, RRID:SCR_012855) was used to develop the API (application programming interface) in PHP language for the application and website.

The Android applications are available on the Play store, and they can be used using Android with minimal version Kitkat (4.4). The memory needed for the TOMO app was 5.3 MB memory, and for the TOMO CM was 5.8 MB memory.

### Workflow of TOMO

**Figure 2.**
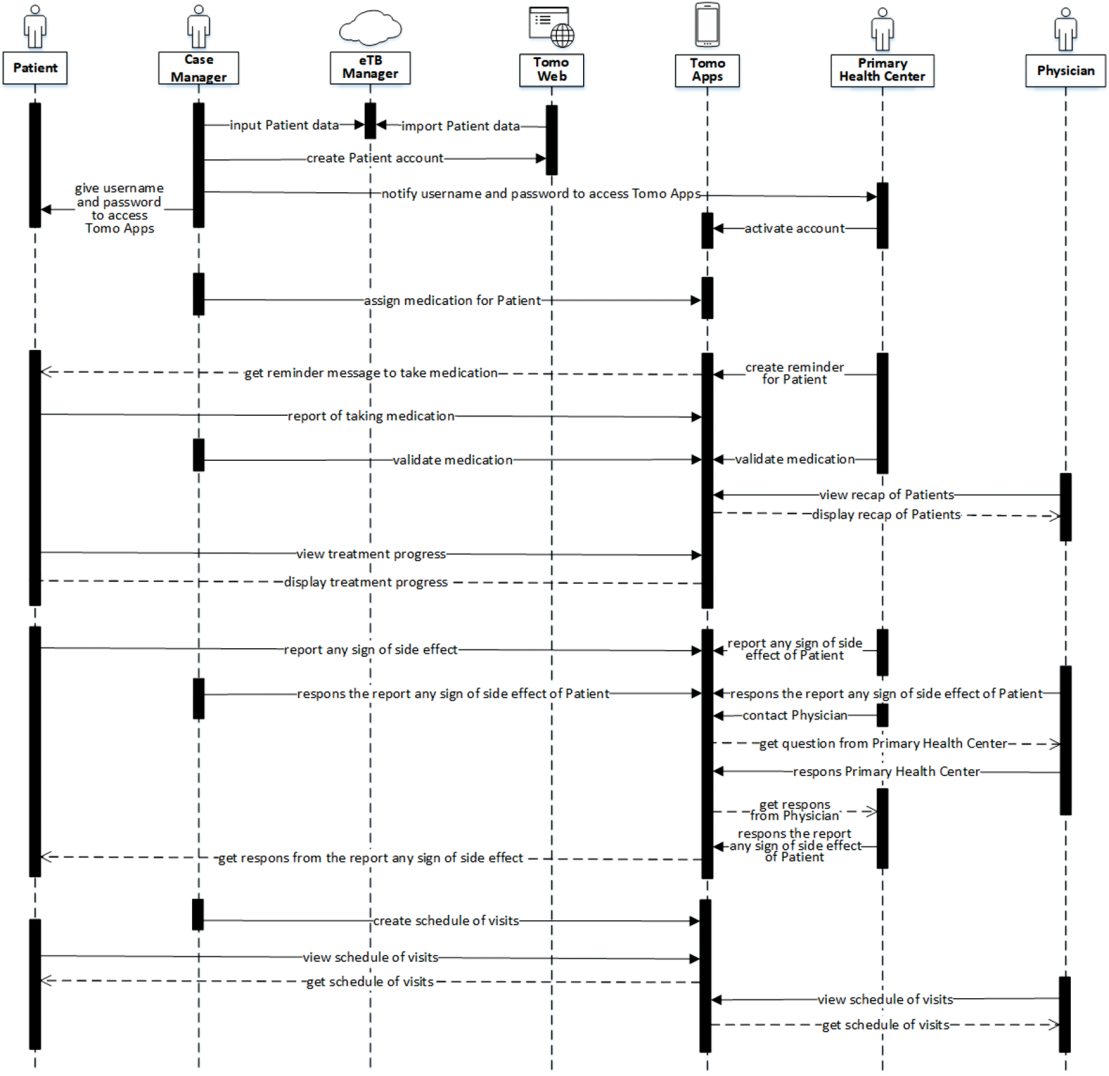
Sequence diagram/workflow of TOMO.

The sequential workflow of the prototype ( Figure 2) shows the process from patient baseline data input, drug consumption monitoring process, adverse event consultation, and the in-person visit schedule feature. Each user type has a different feature in the app. The patient will be responsible for reporting drug consumption and adverse events and for viewing any scheduled visits. The case manager is responsible for inputting patient data and drug regimens, validating drug consumption, responding to patient adverse events, and creating a schedule for physician visits. The primary health center will be mostly responsible for drug consumption validation and reporting patient adverse events. The physician (Pulmonologist) is responsible for evaluating treatment progress and responses to adverse events. The interoperability of the prototype with the existing MDR-TB app was included in the workflow. The patient baseline data will be directly retrieved from the existing app using the API provided by the existing MDR-TB app.

**Figure 3.**
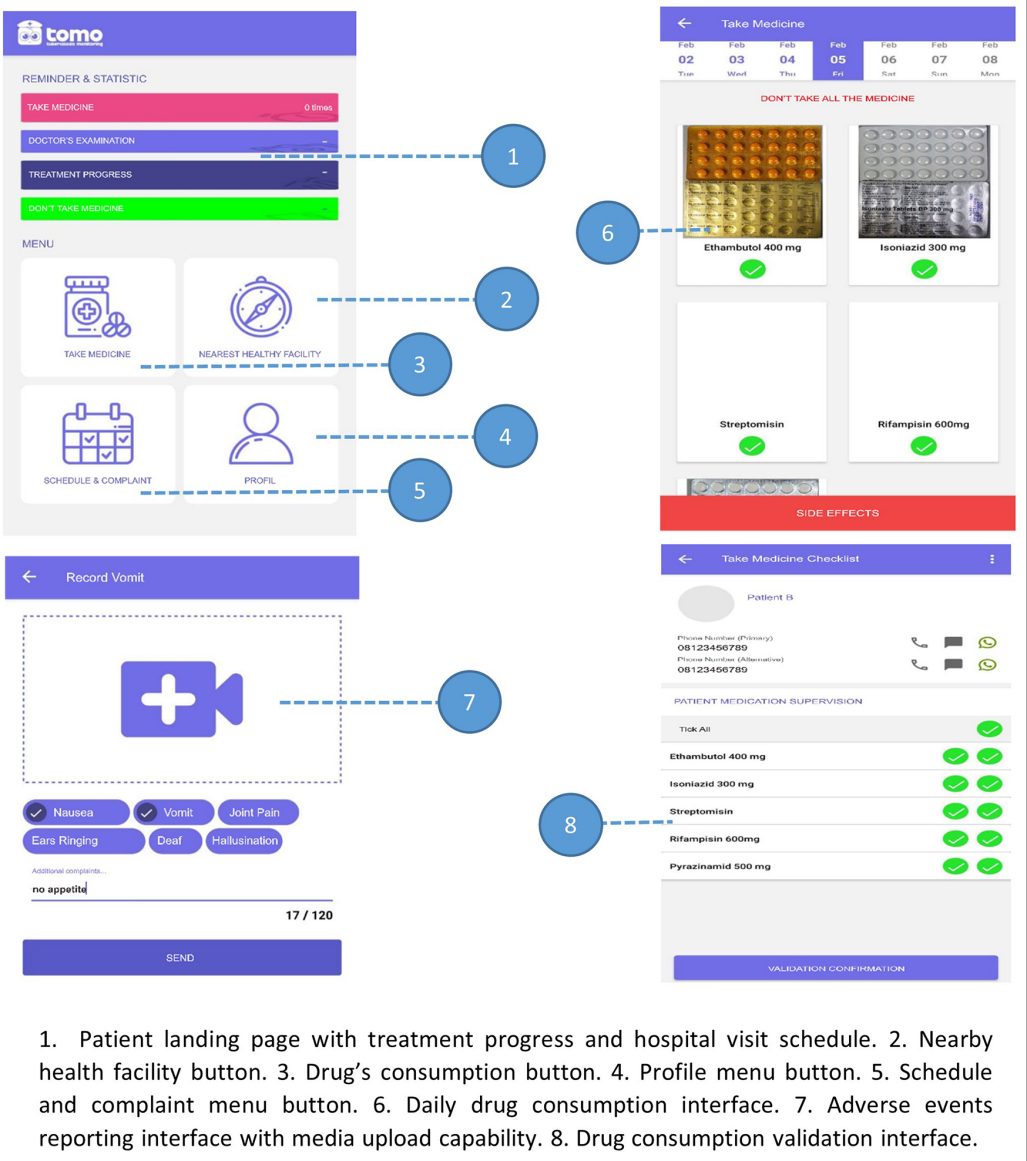
The user interface of TOMO.

The iterative processes of the test were conducted to ensure the performance and feature suitability of the prototype. The app developer conducted alpha and beta testing for bug detection. The simulation by researchers showed that the app functionalities performed well in four different use cases: patients with high adherence, patients with low adherence, patients with adverse events, and patients following treatment in the primary health center without any visit to the MDR-TB center. Two workshops for prototype introduction were conducted by inviting all users. Through the process, the prototype workflow and interface were finalized, and prototype performance was evaluated. The final prototype interface is presented in Figure 3.

## Discussion

This work represents the preliminary design of an app to support the MDR-TB program. The design thinking method in the development process was able to fulfill all user’s needs. In regard to the nine principles of digital development, ^23^ this app development process is inline and covered all aspects of the principles ( Table 2). USAID developed the principle to avoid a failure in the technology-supported program for development. ^23^ The elaboration of the principle and design thinking method allows the MDR-TB app to be more acceptable and sustainable to improve MDR-TB treatment adherence.

**Table 2.** Principles of digital development and TOMO development process.

No	Principles for digital development	TOMO development activities
1	Design with the user	Interview FGD
2	Understand the ecosystem	Desk review, interview, and FGD
3	Design for scale	Stepwise development
4	Build for sustainability	Discussion with local hospital information systems. Consultation with the Sub-directorate of Tuberculosis, Ministry of Health.
5	Be data-driven	Events by users are recorded to develop personalized recommendations, MDR-TB providers used aggregated data for program evaluation.
6	Use open data, open standards, open-source, open innovation	API is still under development. Communication with the MoH has been conducted for data exchange from TOMO with the existing information system.
7	Reuse and improve	Interoperable with existing systems (e-TB Manager) to export some patient demographic data into apps.
8	Address privacy and security	Patients will fill in consent before using the application.
9	Be collaborative	This research involved referral hospitals, health centers, health offices, universities, clinicians and MDR TB management teams, and patients.

Successful experiences from other countries in terms of tuberculosis-related apps were previously reported. In Brazil this consisted of a dedicated Tuberculosis information system within the notifiable disease surveillance system. ^24^ In Peru, a web-based laboratory information system for tuberculosis was reported in improving communication delay in tuberculosis management. ^25, 26^ This work proposes a novel innovation of tuberculosis monitoring by adopting a cross-checking method of drug consumption. Through this method, patient adherence to the treatment and healthcare provider performance as the drug consumption observer could be monitored and evaluated in a real-time fashion. The adverse event consultation session data between patient and healthcare provider were collected in the system and could be used for patient treatment planning in the future.

The study in Brazil proposes web-semantic approach for solving interoperability issues between multiple systems. ^27^ In Indonesia, an initiative has been conducted between TB information systems and hospital information systems. However, a systematic review of the literature revealed that there is a lack of evidence-based incentives for researchers to share data. ^28^ In this regard, an integrated approach has been proposed by Fraser et al. to improve TB information system. This includes open data standards and interoperability, integration with mHealth applications, and ability to function in resource-poor environments. ^29^

## Challenges of app for TB

Mobile phones are the fastest adopted modern technology in developing countries, as has already happened in developed countries. The abilities and features of mobile phones are promising tools in improving community health and health literacy. However, despite the high incidence of mobile phone ownership in the population, the ownership among patients with TB itself is still low. One third of patients with TB in the United States do not have smartphone. ^30^ The situation is similar for patients with hypertension. ^31^ This phenomenon is no different in Indonesia - among poor families one mobile phone is usually used by more than one family member. In addition, as in other developing countries, the ownership of a mobile phone is sometimes not concurrent with the ability to get internet subscription. ^32^ It is thus important for the government to ensure affordable internet access to all communities.

Another challenge is lack of literacy among communities. Many patients with TB come from poor families with low levels of education. ^32^ As mentioned by many studies, lack of literacy is related to poverty and low educational attainment. Furthermore this situation usually leads to inadequate health literacy, which can inhibit the patient’s desire to use novel tools like mobile applications to improve their health. It is important to understand how this particular population interacts with their mobile phone to ensure that the mobile health applications are developed according to these particualr characteristics, so that they are easy to learn and use.

TOMO as a mobile application has a high potential to help patients with MDR-TB in Indonesia. It has been explained in previous studies that patients have lower adherence to completion of treatment when they have had negative treatment experiences. ^33^ These include substantial travel time to get access to care, missed earning time because they have to spend a lot of time at the healthcare facilities, and other expenses by the patients and family who accompanied patient to the facilities. TOMO is developed to be the solution to these difficult situations by functioning as a communication platform which can connect patients directly with their case managers and attending physicians.

## Future directions

We plan to undergo several iterations of evaluation, examining both the usability and content of the app before initiating the trial in a real setting. However, a few improvements are needed based on the real use cases, including modifications of the algorithm. In the near future, data exchange between TOMO with SITB (Sistem Informasi TB) from the Ministry of Health will be exercised. After the implementation of TOMO in Dr. Moewardi Hospital, we will conduct workshops to develop a sustainability plan and initiate scaling up.

Our study has several limitations. Firstly, this evaluation of the use cases was performed in a laboratory setting by the research team members, aiming to validate the functionalities and the system workflow. Secondly, the export/import functionalities were applied based on the former electronic system, namely eTB Manager. Currently, the Ministry of Health has piloted a new electronic TB information system. This new platform offers API functionality offering electronic data exchange of certain variables into our application. We expect interoperability between our prototype with the new system before testing the application in the real setting.

## Conclusion

This report depicts TOMO’s design, technical architecture, and functionalities, an app for improving medication adherence in TB-MDR treatment. This app is a mobile-based platform aimed to improve communication between TB-MDR providers and patients. It contributes to the limited body of literature on improving TB-MDR adherence with digital health interventions, especially using health apps. The prototype features include the adverse event record and report system, reminders for drug taking, and a platform for communication between patients and TB-MDR case managers. The prototype will later undergo several iterations of evaluation for usability and content. Future work will involve developing this into a fully functioning app before initiating the trial in a real setting.

## Data availability

No data are associated with this article.
