# Supplementary material for: Design and prototype of TOMO: an app for improving drug resistant TB treatment adherence
Source: F1000Res. 2021 Sep 29;10:983. [Version 1] doi: 10.12688/f1000research.67212.1 (PMC11372341; doi:10.12688/f1000research.67212.1)
Supplement: Peer review history

# Reviewer report: Reviewer response for version 1

**DOI:** 10.5256/f1000research.70812.r255409

**Published:** 2024-09-03

**Authors:** Malaisamy Muniyandi

**Affiliations:** 1 Department of Health Economics, ICMR-National Institute of Research in Tuberculosis(NIRT), Chennai, India

**Manuscript number **F1KR00CDE F1R-VER70812-R

**Title:** ****Design and prototype of TOMO: an app for improving drug resistant TB treatment adherence

**Overall comments:** This manuscript focused on an important topic an app for improving drug resistant TB treatment adherence and presented the sequential steps to develop the app, its general architecture, and its functionalities.  Overall this manuscript address important issue and well written paper I recommended for indexing with minor revisions and worth emphasising the following points.

**Specific comments **

- The authors mentioned that already few m-Health applications exits for TB.  It will be useful for the reader, provided the details on what is novel in this app or any new features added in this app.
- What is the treatment success rate if we use this app to increase treatment adherence for MDR-TB
- What way it is different than the 99DOTS, MERM boxes,
- What is the recommendation for us of this app to other countries to increase MDR-TB treatment adherence.
- Is it only for MDR-TB; What about other TB patients for this app  
- Conclusion to be based on this current study findings. The conclusion provided in this paper is not  from this study findings

(TOMO app could be used by patients, primary health centers, clinical teams, and case managers……)

- This is the good work, needs further for implementation to see the feasibility and acceptability of this app for MDR-TB patients
- Minor edits required
  - Background: ‘multi drugs tuberculosis’ to be changed as  ‘multi drug resistance tuberculosis’
  - TB-MDR to be changed as MDR-TB
  - The terms used are m-Health, mobile health, e-TB manger, mHealth is these are same, or what is context authors describing these terms. If these are same then the same terminology to be used throughout the text.
  - Need to provide references for:  Page-3 Para 5: line 2

A comprehensive review of the potential for digital health in TB programmes has been published.

-
  - Page-9 Para 1: line 1

Successful experiences from other countries in terms of tuberculosis-related apps were previously reported.

-
  - Page-9 last Para: line 2

As mentioned by many studies, lack of literacy is related to poverty and low educational attainment

-
  - Abbreviation TB to followed throughout text
  - Conclusion to changed; it seems to be developed the app, it needs to checked under the field condition.
- What about cost-effeteness of this intervention

 Overall it is a good work needs revision considering the above comments.

Is the work clearly and accurately presented and does it cite the current literature?

Yes

If applicable, is the statistical analysis and its interpretation appropriate?

Not applicable

Are all the source data underlying the results available to ensure full reproducibility?

No source data required

Is the study design appropriate and is the work technically sound?

Yes

Are the conclusions drawn adequately supported by the results?

No

Are sufficient details of methods and analysis provided to allow replication by others?

Partly

Reviewer Expertise:

Health Economics, HTA, TB

I confirm that I have read this submission and believe that I have an appropriate level of expertise to confirm that it is of an acceptable scientific standard.

# Reviewer report: Reviewer response for version 1

**DOI:** 10.5256/f1000research.70812.r255412

**Published:** 2024-04-23

**Authors:** Wilson Tumuhimbise

**Affiliations:** 1 Mbarara University of Science and Technology, Mbarara, Western Region, Uganda

Thank you for the opportunity to review this manuscript. I have noted a few areas that require clarification.

 Methods

 How many collaborators were involved. Its wonderful to be specific about the exact number of collaborators that were part of this phase. This should be revised in the entire manuscript so that specificity and clarity and ensured.

 There is a need to ensure consistency in wording, in some sections, you mention meetings while in others you mention workshops, these should be revised. Ideally it would be great to mention the composition of these meetings, how many members made up each of these meetings, how were they selected, what was the eligibility criteria, this needs to be clear and how was data from these meetings obtained, was it through recordings and later transcribed verbatim.

 Health apps, deal with a huge amount of sensitive health data, how is the security of this app ensured given that unintended disclosure of health status has been cited in some literature.

 Is this app available free of charge or it requires participants to pay a certain fee additionally, does it rely on the internet to function, or it has an offline version?

 Provide the section of strengths and limitations of this study.

Is the work clearly and accurately presented and does it cite the current literature?

Yes

If applicable, is the statistical analysis and its interpretation appropriate?

Not applicable

Are all the source data underlying the results available to ensure full reproducibility?

Partly

Is the study design appropriate and is the work technically sound?

Yes

Are the conclusions drawn adequately supported by the results?

Partly

Are sufficient details of methods and analysis provided to allow replication by others?

Yes

Reviewer Expertise:

digital health

I confirm that I have read this submission and believe that I have an appropriate level of expertise to confirm that it is of an acceptable scientific standard, however I have significant reservations, as outlined above.

# Reviewer report: Reviewer response for version 1

**DOI:** 10.5256/f1000research.70812.r255407

**Published:** 2024-04-15

**Authors:** Ellen Mitchell

**Affiliations:** 1 Department of Public Health, Institute of Tropical Medicine, Antwerp, Belgium

**TOMO App Review**

 Thank you for the opportunity to review this manuscript about the sequential development and piloting of a mobile app designed to facilitate communication among parties in the DR-TB treatment process. There are strengths, areas for further elaboration, and questions about the manuscript that are summarized as follows:

**Strengths **

1. The authors are to be commended for the thoughtful, step-wise process described in this manuscript.
2. This manuscript will be useful for those seeking to replicate the process in other contexts. The key features of interoperability, trialability, automatic aggregation and dashboard, seem very good for lowering the work load of busy health care workers and program staff.
3. Documenting patient reported outcomes(PRO) is so important and the DR-TB field is so far behind in this area, that it is great to see pharmacovigilance incorporated in a digital health tool. While some might argue that this is shifting the recording and reporting AE burden from the clinician to the patient, others may see it as providing a forum for patients to voice their discomfort and suffering in real time. The question then  becomes: what, if any remedial actions are taken in response to the call for help.  We must all stay tuned for the next paper to find out. It is only empowering if the call for help is answered with support and solutions.

**Areas for further refinement**

1. The authors suggest at the beginning that adverse drug events are the main driver of non-persistence on treatment. Certainly, side effects, symptoms of the disease, stigma, catastrophic costs, low quality care, and other barriers also play a role in early withdrawal from a treatment course. Later on in the manuscript is this statement “ [drivers *] include substantial travel time to get access to care, missed earning time because they have to spend a lot of time at the healthcare facilities, and other expenses by the patients and family who accompanied patient to the facilities. TOMO is developed to be the solution to these difficult situations by functioning as a communication platform which can connect patients directly with their case managers and attending physicians*.”  This sentences seems to imply that if they use the app they don’t need to go to the health facility daily, that the self-reported self administration of treatment (SAT) relieves them of this obligation. Is that correct? Is this a replacement for DOT? That is inferred but not explicitly stated. If it is not a form of SAT, then how exactly does TOMO solve the substantial travel time and missed earning time issue?
2. It would be good to have a figure or a mapping of what the main reasons for forsaking treatment are (from the patient survey) and how the app’s features specifically corresponds to these.  
3. There seems to be a disconnect at times between what is expressed as a need and what is provided as a service. From the survey, MDR patients report needing social support. The notion that an unmoderated chat group of MDR-patients would provide support and increase adherence is interesting, but I am unaware of the evidence to support this claim. Consider to offer some evidence to  motivate the decision to have an unmoderated chat at the support intervention . Unmoderated chat groups can be used in many ways by their users. It would be good to know if the group is anonymous or not. Anonymous chat groups of vulnerable groups have not had a great track record. For example, peer-support apps for opioid disorder often contain misinformation, trolling, and harassment. How will you ensure that people with TB are not harmed in the group chat?
4. The authors point out that there is a lot of enthusiasm for digital health for TB adherence. However, it would be good to point out that the results of digital health trials for modifying behavior of people with TB have been mixed.  Qualitative work suggests that patients value **the human element **in their care and are not necessarily keen to interact with robots, electronic pillboxes, SMS messages.

**Comment regarding the App development (not the manuscript)**

1. The TOMO app seems to have been co-designed with pulmonologists and primary care workers. The MDR patients are surveyed at the beginning, and they are involved in testing the prototype, but the middle part of *the actual design of the app features* does not include them. Digital health for end users with TB that is not co-designed by people with TB can lead to them feeling even further alienated from society. It appears that health care workers are the main beneficiaries of this system as currently designed.

**Areas that are not yet clear to this reader:**

1. Access to smart phones is lower among women and poorer persons.  The provision of support and other benefits via smart phones and the requirement of having a bank account disadvantages women, those under 18, and the poor. Lack of electricity, internet connectivity; and low technology literacy are also more common among the poor. How can the app be modified to contribute to equity? How do the 31% without a smart phone differ from those with a smart phone?
2. While reporting adverse events is important for the program, it is less clear how this is useful for the patient, unless there is a change in regimen or offer of symptom management. Does the app measure *whether and how long it takes* for there to be a clinical response? Are the reminders to respond also pinging the HCWs? Or just the patients?

Is the work clearly and accurately presented and does it cite the current literature?

Partly

If applicable, is the statistical analysis and its interpretation appropriate?

Not applicable

Are all the source data underlying the results available to ensure full reproducibility?

No source data required

Is the study design appropriate and is the work technically sound?

Partly

Are the conclusions drawn adequately supported by the results?

Yes

Are sufficient details of methods and analysis provided to allow replication by others?

Partly

Reviewer Expertise:

TB

I confirm that I have read this submission and believe that I have an appropriate level of expertise to confirm that it is of an acceptable scientific standard, however I have significant reservations, as outlined above.
